# Analyzing international medical graduate research productivity for application to US neurosurgery residency and beyond: A survey of applicants, program directors, and institutional experience

**DOI:** 10.3389/fsurg.2022.899649

**Published:** 2022-07-27

**Authors:** Giancarlo Mignucci-Jiménez, Yuan Xu, Lena Mary Houlihan, Dimitri Benner, Jubran H. Jubran, Ann J. Staudinger Knoll, Mohamed A. Labib, Teodoro Forcht Dagi, Robert F. Spetzler, Michael T. Lawton, Mark C. Preul

**Affiliations:** ^1^ The Loyal and Edith Davis Neurosurgical Research Laboratory, Department of Neurosurgery, Barrow Neurological Institute, St. Joseph’s Hospital and Medical Center, Phoenix, AZ, United States; ^2^ Mayo Medical School, Rochester, MN, United States

**Keywords:** international medical school graduates, neurosurgery research, neurosurgery residency, neurosurgery residency application, research productivity

## Abstract

**Background:**

The authors investigated perceived discrepancies between the neurosurgical research productivity of international medical graduates (IMGs) and US medical graduates (USMGs) through the perspective of program directors (PDs) and successfully matched IMGs.

**Methods:**

Responses to 2 separate surveys on neurosurgical applicant research productivity in 115 neurosurgical programs and their PDs were analyzed. Neurosurgical research participation was analyzed using an IMG survey of residents who matched into neurosurgical residency within the previous 8 years. Productivity of IMGs conducting dedicated research at the study institution was also analyzed.

**Results:**

Thirty-two of 115 (28%) PDs responded to the first research productivity survey and 43 (37%) to the second IMG research survey. PDs expected neurosurgery residency applicants to spend a median of 12–24 months on research (Q_1_-Q_3_: 0–12 to 12–24; minimum time: 0–24; maximum time: 0–48) and publish a median of 5 articles (Q_1_-Q3: 2–5 to 5–10; minimum number: 0–10; maximum number: 4–20). Among 43 PDs, 34 (79%) ranked “research institution or associated personnel” as the most important factor when evaluating IMGs' research. Forty-two of 79 (53%) IMGs responding to the IMG-directed survey reported a median of 30 months (Q_1_-Q_3_: 18–48; range: 4–72) of neurosurgical research and 12 published articles (Q_1_-Q_3_: 6–24; range: 1–80) before beginning neurosurgical residency. Twenty-two PDs (69%) believed IMGs complete more research than USMGs before residency. Of 20 IMGs conducting dedicated neuroscience/neurosurgery research at the study institution, 16 of 18 who applied matched or entered a US neurosurgical training program; 2 applied and entered a US neurosurgical clinical fellowship.

**Conclusion:**

The research work of IMGs compared to USMGs who apply to neurosurgery residency exceeds PDs' expectations regarding scientific output and research time. Many PDs perceive IMG research productivity before residency application as superior to USMGs. Although IMGs comprise a small percentage of trainees, they are responsible for a significant amount of US-published neurosurgical literature. Preresidency IMG research periods may be improved with dedicated mentoring and advising beginning before the research period, during the period, and within a neurosurgery research department, providing a formal structure such as a research fellowship or graduate program for IMGs aspiring to train in the US.

## Introduction

Professional neurosurgery organizations in the United States (US), such as the American Association of Neurological Surgeons (AANS), Neurosurgery Research and Education Foundation, Congress of Neurological Surgeons, American Board of Neurological Surgery, Society of Neurological Surgeons, and various regional and state associations, continue to advocate for dedicated research time during neurosurgical training, with a full year of research designated within the residency structure. The founding departments of US neurosurgery training programs have been, and continue to be, leaders in neurosurgery and neuroscience research within the US. This trend continued as most other neurosurgery departments and training programs were established. Thus, in addition to developing training technology, the human factor of performing research continues to be of major importance for neurosurgery education and residency applicants to US programs, especially in the ever-more connected international world of neurosurgery. Indeed, applicants may spend years in research before residency, involving significant professional and life planning. International medical graduates (IMGs) with an outlook toward US neurosurgery residency in the next 10 years may already be involved in such career decisions.

The question that follows is, “Why do IMGs apply for neurosurgery training in the US?” The answer is multifactorial, but it can be inferred that the IMGs believe that either the training or quality of life in the US is superior to that of their home country. Previous papers have focused on IMGs' perceptions of neurosurgery residency in their own country, especially in low to middle-income countries (LMICs). Deora et al. (1) sent a questionnaire through social media to all neurosurgical residents in LMICs, asking general questions about their perspectives on their training programs. Significant differences between US and LMIC residency programs were found in work-hour regulations and subspecialty training. Substantial gaps in residency experience were noted; 40% of respondents did not report substantial residency experience in any of the queried subspecialties (i.e., endovascular, epilepsy, deep-brain stimulation/lesioning, minimally invasive surgery, radiosurgery, or deformity surgery). The lack of subspecialty training in a candidate's respective country could be a major factor in their decision to pursue US-based training. The US training system is perceived as organized, complete, and accepting of IMGs. The training programs in LMICs are inherently limited due to local, geographical, infrastructure, and economic factors (2).

In a 2018 study, IMGs represented 24% of the US physician workforce and 1 in 4 trainees in US residency programs (3). These numbers are likely to rise in the coming years as major physician shortages develop due to increased health care demand, workforce shortages due to the recent COVID-19 pandemic and government mandates, and an aging workforce. The projected US physician deficit is 139,160 by 2030. A well-recognized shortage of neurosurgeons is likely to increase similarly, as 46% of practicing neurosurgeons are 55 years of age or older (4, 5).

IMGs account for 13% of practicing physicians, 6% of neurosurgical residents (8% in 2018), and 11% of academic neurosurgeons (3, 6, 7). The need for neurosurgeons is met by USMGs and IMGs, which reflects the competitive nature of the neurosurgery residency match, with 66.8% (211/316) and 65.2% (211/322) of allopathic USMGs matching in 2020 and 2021, respectively. In contrast, 28.6% (18/63) and 25.8% (17/66) of IMGs matched in 2020 and 2021, respectively, as reported by the National Resident Matching Program (NRMP) (8, 9). Among IMGs, 25% (12/48) and 22% (11/50) were non-US IMGs in the 2020 and 2021 match cycles, respectively. Overall, USMGs, including graduates from both allopathic and osteopathic medical schools, comprised 92.2% (214/232) and 92.7% (217/234) of applicants who matched in 2020 and 2021, respectively. Conversely, US and non-US IMGs comprised only 7.8% (18/232) and 7.3% (17/234) of matches (8, 9). These numbers show the discrepancy between USMGs and IMGs.

Neurosurgery is uniquely intertwined with scientific work, and an overwhelming majority of training programs are affiliated with major academic institutions. This characteristic contributes to neurosurgery applicants having the highest research productivity of all medical specialties in the US (10). IMGs seeking to overcome the difficulty of matching with a US neurosurgical residency program view high-level research as critical to overcoming this difficulty. IMGs perceive higher h-indices and numbers of published articles as an advantage for matching with a US program (11).

Nonetheless, studies have reported biases affecting IMGs in the US neurosurgical matching system (7, 11, 12). Sheppard et al. (12) reported that IMGs are more likely to match at unranked or lower-ranked residency programs compared to USMGs despite high research output, publications, and the research impact. The likelihood of a USMG vs. an IMG matching into a ranked program was almost 3 times higher (OR = 1.7 vs. 0.59). Khalafallah et al. (7) conducted a retrospective review of 2,749 residents spanning 50 years. They reported that IMGs were significantly more likely than USMGs to have completed a research fellowship after medical school and before residency (16% vs. 2%). Chandra et al. (11) reported that from 2009 to 2017, the number of IMG applicants increased without a significant increase in submitted applications or matched IMGs over this period. These individual findings reveal that research productivity is important for matching into a neurosurgery training program. However, IMGs are still limited in their acceptance into a ranked training program (e.g., *U.S. News & World Report* “Best Hospitals for Neurology & Neurosurgery” ranking) (13).

Although the geographical location where IMGs received graduate education and the characteristics of success in their neurosurgical match have undergone recent analysis (6, 11), an investigation into IMGs' neurosurgical research, coupled with the program directors' (PDs) expectations, has yet to be reported. Neurosurgical and basic science laboratories of neurosurgery departments are the mainstay of departmental research productivity and commonly host postdoctoral researchers from home and abroad. We obtained successful IMG matching data for those who conducted dedicated research in our institution's neurosurgical laboratory.

Some researchers have accessed publicly available databases to analyze broad trends and outcomes for IMGs applying to neurosurgery residency programs, which have required large sample sizes (7, 11, 12). However, we desired a more focused and granular study of the features of a successful IMG application to residency programs. We sought to assess the research productivity of IMGs—both from their perspective and that of PDs—using direct, anonymous surveys to understand the personal aspects of researchers that cannot be ascertained from publicly available databases. For this study, IMGs comprise all individuals who received medical degrees outside the US, irrespective of their nationality. This survey was limited to the most recent 8-year span (July 2013 through June 2020) of IMGs currently or recently matched in US neurosurgery residency programs and a separate survey encompassing all 20 IMG neurosurgery research fellows from our institution who applied and were successfully matched into neurosurgery residency programs or who entered neurosurgery clinical fellowships. The findings elucidate the key research period-related components of a successful IMG application and compare the research experience of IMGs with that of USMGs who successfully matched with US-based neurosurgery programs.

## Materials and methods

### Data collection

No protected health information and no individually identifiable information were collected. No patients were involved in this study. Therefore, no institutional review was sought or required.

A search for all neurosurgical residency training programs in the Directory of the AANS and the Association of American Medical Colleges (AAMC) for the 2020–2021 match cycle revealed 115 training programs. Every PD identified through the AANS directory (14) was provided a survey including qualitative and quantitative questions, focusing on all applicants, their experiences with IMGs in a research environment, and how IMGs relate to USMGs. Later, every PD was contacted again and provided an additional survey, focusing on the importance of different factors associated with an IMG applicant's research productivity.

All Accreditation Council for Graduate Medical Education–approved neurosurgery residency programs in the US listed by the AAMC for the 2020–2021 cycle were identified using the AAMC's online portal (15). All programs older than 7 years (i.e., had graduated at least 1 resident) were then identified, and individual public websites were reviewed for the most updated list of current residents. An IMG was defined as any resident who had completed his or her primary medical degree (MD, MBBS, MBChB, or others) at a medical college outside of the United States. In addition, public residency websites of all identified programs were reviewed for up-to-date information on their current residents. Residents who received their medical degrees abroad were identified, and publicly available information was collected. A search before and after residency graduation in June 2020 revealed 8 residency classes and 79 IMGs. A questionnaire with both qualitative and quantitative questions on their research experiences before and after the neurosurgical match was sent to the 79 IMGs.

Data regarding IMGs who successfully matched in either a US residency program (i.e., neurosurgery or other) or entered a clinical fellowship program and conducted a dedicated research period at Barrow Neurological Institute (Barrow) from 2000 through 2020 were collected with the permission of the Director of Neurosurgery Research at Barrow. All IMGs completed a research fellowship, and some completed an additional integrated interdisciplinary neuroscience PhD program. Research program type, research focus, match specialty (i.e., neurosurgery, other, or clinical fellowship), months of research, geographical region, h-index, and the number of publications associated with Barrow before residency, 1 year after matching, and 2 years after matching into a residency program were collected and analyzed. A retrospective bibliographic search was done for these Barrow IMGs using PubMed. A publication was added to their total count if the IMG was the first author or co-author and the publication was associated with Barrow. The Scopus author profile database (16) was used to determine each author's h-index.

### Survey content

The first PD and IMG surveys were delivered between April 2020 and January 2021. The second PD survey was delivered in April 2022. The first PD survey contained 5 questions that assessed: (1) the number of years PDs believe IMGs should spend on research before their residency application; (2) the number of peer-reviewed publications any neurosurgery residency applicant should have published; (3) whether PDs believe IMGs complete more research than USMGs before residency; (4) whether PDs believe IMGs complete more research than USMGs during residency, and (5) whether PDs believe nonresident research fellows (i.e., full-time research fellows) or residents were more productive in scholarly research than US research fellows and residents if their program supported such research programs. The second PD survey contained 1 question, asking the PD to rank from 1 to 4 the importance of the following factors when evaluating an IMG applicant's research productivity: (1) the research institution or associated personnel, (2) the impact of the research, (3) the number of publications, and (4) a structured research period or theme.

The IMG survey contained 12 questions. Two were demographic assessments of sex and country of origin—the country of origin was later categorized as a geographical region (i.e., North America, South America, Europe, Middle East, North Africa, South Africa, South Asia, and East Asia) to protect the identity of residents. Three questions inquired whether the IMG spent time on research in a neurosurgery laboratory, the length of their neurosurgery research experience, and whether they spent time in more than one laboratory. The remaining 7 questions dealt with (1) their training background before obtaining a neurosurgical residency, (2) their motivation in seeking neurosurgery research, (3) the degree this research impacted their future career, (4) if they would recommend dedicated research time to peers and future applicants, (5) what their current position was at the time of the survey, (6) how many papers they published before residency, and (7) how many papers they published after beginning residency.

### Analysis

Collected respondent survey data was stored on a password-protected computer and backed up on an encrypted drive. Data of respondents were given an anonymizing number code for identification. Qualitative answers were reported in full whenever their content diverged from others in a meaningful way. Specific sample means in the PD and IMG-directed surveys were analyzed. Data were expressed as medians with first and third quartile ranges (Q_1_-Q_3_) and absolute ranges and then compared using the Mann-Whitney test. The means, medians, and minimum and maximum responses of the PDs were compared to the responses of the IMGs. GraphPad Prism version 9.3.1 (GraphPad Software, San Diego, California, USA) and Microsoft Excel version 16.58 (Microsoft Corporation, Redmond, Washington, USA) were used for data analysis.

## Results

### Program directors

**Figure 1:**
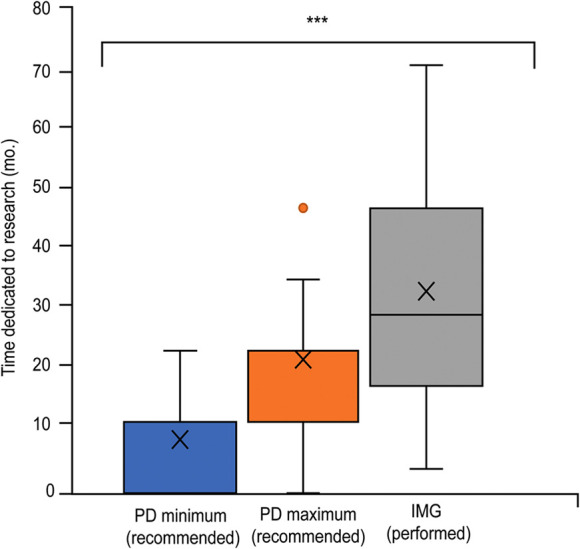
Box and whisker plot of the length of dedicated research (months) completed by international medical graduates (IMGs) before matching into neurosurgical residency, compared to the expectations neurosurgical residency training program directors (PDs) have of applicants. The X represents the mean. The horizontal line (if visible) represents the median. Whisker lines represent the maximum and minimum values. Additional dots represent outliers that did not fit the model. (***) Represents statistical significance (*P* < 0.05). *Used with permission from Barrow Neurological Institute, Phoenix, Arizona*.

**Figure 2:**
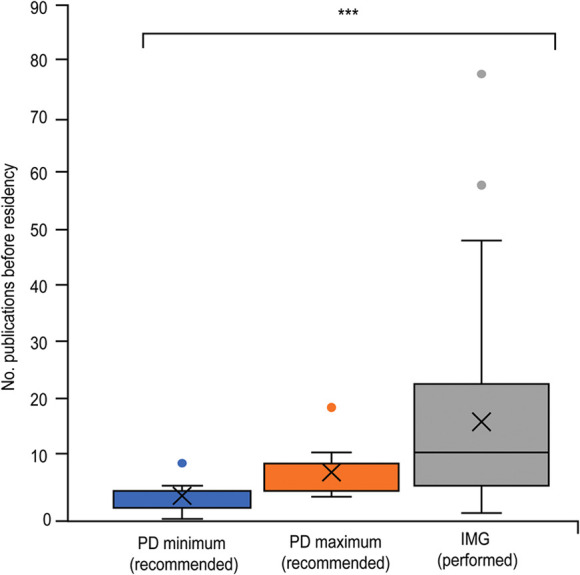
Box and whisker plot of the number of publications of IMGs before matching into neurosurgical residency, compared to PD applicant expectations. The X represents the mean. The horizontal line (if visible) represents the median. Whisker lines represent the maximum and minimum values. Additional dots represent outliers that did not fit the model. (***) Represents statistical significance (*P* < 0.05). *Used with permission from Barrow Neurological Institute, Phoenix, Arizona*.

Of 115 programs contacted, we received 32 nearly complete responses (28%) to the first PD survey. These PDs responded that neurosurgery residency applicants should spend 12 to 24 months (Q_1_-Q_3_: 0–12 to 12–24; minimum range, 0–24, maximum range, 0–48 months) on research (Figure 1). They also expected the applicants to have published a median of 5 articles (both minimum and maximum medians = 5) (Q_1_-Q_3_: 2–5 to 5–10; minimum range 0–10, maximum range 4–20) before applying (Figure 2). Two PDs stated that the answer to both questions was variable. One suggested taking additional factors into account when evaluating the research capabilities of applicants, such as the research opportunities their medical school offered. Furthermore, 22 (69%) PDs answered that IMGs completed more research than USMGs before residency. When asked whether IMGs are engaged in more research than USMGs once they enter residency, 11 (34%) PDs answered yes. In comparison, 14 (44%) PDs believed that IMGs were not more productive during residency, and 2 (6%) emphasized the IMG's character rather than the residents' respective medical school location. Seventeen (53%) PDs stated that their program regularly supports research fellows, 10 (31%) PDs responded that research fellows are more productive than residents, while 3 (9%) assessed residents to be more productive than full-time research fellows. Four others (13%) suggested generalization is impossible or that IMGs' and USMGs' productivity did not differ.

In response to the second PD survey of 115 programs, 43 (37%) PDs provided complete responses. Thirty-four of the 43 (79%) PDs responded that the prestige or reputation of the research institution or associated personnel was the most important factor when evaluating an IMG's research productivity. Nine (21%) PDs responded that the impact of research was the most important factor. Twenty-eight (65%) PDs responded that a structured research period or pursuing a thematic research topic was the third most important factor. All 43 (100%) PDs responded that number of publications is the least important factor when evaluating an IMG applicant's research productivity (Table 1).

**Table 1:** Neurosurgical residency training program director (*n* = 43) ranking of research productivity evaluation factors.

Factor	Program director ranking^a^ *n* (%)			
	1	2	3	4
Research institution or associated personnel	34 (79)	9 (21)	0 (0)	0 (0)
Impact of research^b^	9 (21)	19 (44)	15 (35)	0 (0)
Structured research period or theme^c^	0 (0)	15 (35)	28 (65)	0 (0)
Number of publications	0 (0)	0 (0)	0 (0)	43 (100)

^a^ Ranking: most important (1) to least (4) important.

^b^ For example, impact factor of journal or h-index.

^c^ For example, graduate program, multiple projects covering same topic.

### International medical graduates

**Figure 3:**
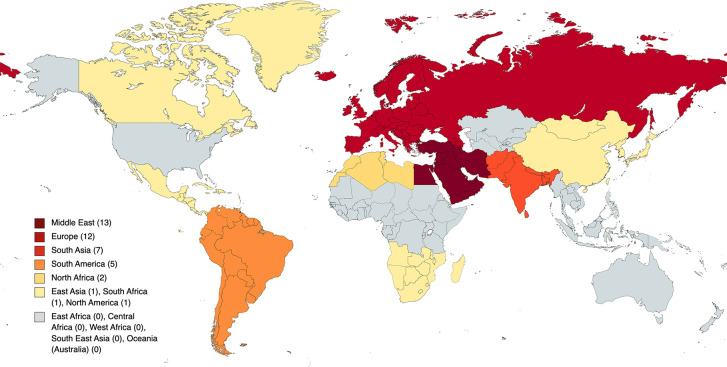
Geographic data of IMGs’ home countries by region. Small countries of origin were categorized into regions to protect the identities of IMG residents who come from such countries. *Copyright made available under the Creative Commons Attribution-ShareAlike 4.0 International (CC BY-SA 4.0) (*https://creativecommons.org/licenses/by-sa/4.0).

Responses came from 42 of 79 (53%) residents contacted for the IMG-directed survey. Of those 42, 13 (31%) were from the Middle East, 12 (29%) from Europe, 7 (17%) from South Asia, 5 (12%) from South America, and 2 (5%) from North Africa; 1 (2%) each was from North America, South Africa, and East Asia (Figure 3). Thirty-nine (93%) respondents were men, and 3 (7%) were women. All respondents participated in resolute neurosurgical or neuroscience research before their match. Twelve (29%) spent time in multiple research laboratories.

Asked about the degree to which their research in a neurosurgical laboratory impacted their future career, 26 (62%) IMGs responded with “A great deal,” and the other 16 (38%) with “A lot.” A total of 35 (83%) would recommend dedicated research time to their peers and future neurosurgery applicants, whereas 7 (17%) would not. Their respective year in training (i.e., postgraduate year) was removed from analyses to protect the identity of each respondent. Among the 42 IMGs, 11 (26%) completed a neurosurgery residency training program abroad, and 14 (33%) attended foreign postgraduate training without completing a neurosurgical residency (4 with incomplete neurosurgical training, mandatory rural service, master's degree, or surgical internship). In contrast, 17 (40%) received no postgraduate training before coming to the US to apply to a residency program (Figure 2).

**Figure 4:**
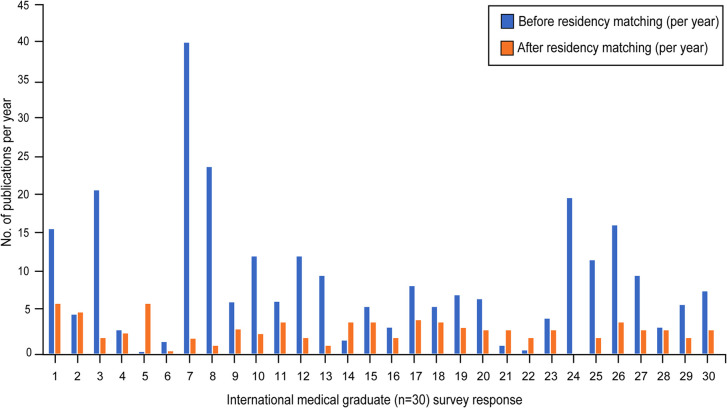
Graphical representation of the number of publications per year of IMG residents that were completed before (i.e., during their dedicated research time, *blue*) and after matching into a neurosurgical residency (*orange*). Residents entering postgraduate-year 1 and those who chose not to answer the question were excluded from this analysis. *Used with permission from Barrow Neurological Institute, Phoenix, Arizona*.

When asked about the primary motivation for their research work, 12 (29%) IMGs stated that they wanted to improve their chances of a neurosurgical match, and the remaining 30 (71%) commented on their passion for neurosurgical research. Before beginning their neurosurgical residency, 42 IMGs reported a median of 30 months (Q_1_-Q_3_: 18–48; range 4–72 months) spent in neurosurgical research and 12 published articles (Q_1_-Q_3_: 6–24.3; range 1–80), with 1 vacant answer (Figures 1, 2). Thirty IMGs reported their research productivity before and after successfully matching into residency (Figure 4). The number of publications differed significantly before and during residency (*P* < 0.001). Of the 30 IMGs reporting this information, 25 (83%) had more publications before than during residency. The median number of publications per year for an IMG before matching was 6.8 (Q_1_-Q_3_: 3.3–12.5; range 0.2–40), while the median number per year for an IMG during residency was 3 (Q_1_-Q_3_: 2–4; range 0–6.4). Only 5 of 30 (17%) IMGs published more articles after entering residency (Figure 4).

### Comparison between PD recommendations and IMG performance

The minimum and maximum recommendations by PDs for months of research and the number of publications before matching were combined and compared to actual IMG performance for each variable. The PDs' median recommended research period was 18 months, and the median recommended number of publications was 5. The median IMG-performed months of research (30 months) and the number of publications (12) before matching were both significantly larger than the recommended PD values (*P* < 0.001 and *P* < 0.001, respectively).

### Barrow IMG matching results

Twenty IMGs who spent dedicated research time at Barrow during the study period were evaluated (Table 2). All 20 completed a named neurosurgery research fellowship of the hospital institution. In addition, 3 (15%) of these IMGs were part of a nationally-ranked integrated interdisciplinary neuroscience PhD program with a local major state university. Sixteen (80%) Barrow IMGs applied to US neurosurgical residency programs, 2 (10%) applied for residency in another medical specialty, and 2 (10%) applied for US neurosurgery clinical fellowships. All 20 IMGs matched or entered the specialty of their choice. All 3 IMGs who completed the PhD program were matched into a US neurosurgery residency program. Three (15%) IMGs were accepted into a residency upon their second application, with two continuing a research fellowship in the meantime. Another was accepted into a preliminary general surgery year and was then admitted 2 years later into a vacated neurosurgery position. Four (25%) of the 16 IMGs who applied to neurosurgery were accepted into the Barrow neurosurgery residency. However, the other 12 (75%) were all admitted to competing top neurosurgery residencies.

**Table 2:** Characteristics and research data of international medical graduates (IMGs) who completed a dedicated research period at Barrow Neurological Institute.

IMG	Research		Match Specialty	Research Time (mo.)	Barrow Publications				h-Index^b^	Region
	Type	Focus			Total^a^	Before Residency	+1 Y	+2 Y		
1	Fellow	SA	NS	24	31	3	3	2	43	South America
2	Fellow	SA	NS	36	7	4	0	2	9	South America
3	Fellow	ET	CF	24	1	1	0	0	6	East Asia
4	Fellow	SA, SBM	NS	24	45	9	3	4	22	Europe
5	Fellow	SCI, BI	NS	24	4	4	0	0	10	North America
6	Fellow	SCI	NS	36	2	0	0	0	23	Europe
7	Fellow	SA	CF	36	30	11	4	1	15	South America
8	Fellow	SBM	NS	24	22	0	2	5	14	North America
9	Fellow	SA	Other	12	2	0	0	0	2	North America
10	Fellow + PhD	FT, SCP	NS	48	58	12	2	6	21	Europe
11	Fellow	ST	NS	12	3	2	0	0	13	South Asia
12	Fellow	SCI, BTI	Other	24	10	4	2	1	11	Europe
13	Fellow + PhD	SA	NS	36	20	8	7	5	13	Middle East
14	Fellow	SA	NS	24	46	0	0	4	9	Middle East
15	Fellow	SA	NS	48	103	88	11	4	8	Middle East
16	Fellow	FT, SA	NS	25	39	28	9	0	11	Europe
17	Fellow	SA	NS	24	59	49	9	1	9	East Asia
18	Fellow + PhD	FT, ST	NS	70	91	80	10	1	17	Europe
19	Fellow	VNTs	NS	24	60	60	0	0	12	South Asia
20	Fellow	SA	NS	24	42	42	0	0	20	Middle East

Abbreviations: BI, brain imaging; BTI, brain tumor immunology; CF, clinical fellowship; ET, endovascular technology; FT, fluorescence technology; NS, neurosurgery; SA, surgical anatomy; SBM, spine biomechanisms; SCI, spinal cord injury; SCP, spinal cord physiology; ST, surgical technology; VNTs, varied neurosurgical topics.

^a^ Total number of publications associated with Barrow, even after completion of a dedicated research period.

^b^ The Scopus author profile database was used to determine the h-index. This includes publications not associated with Barrow and those published more than 2 years after matching.

For all IMGs at Barrow, the median number of research months was 24 (Q_1_-Q_3_: 24–36; range: 12–70). The median number of total publications was 31 (Q_1_-Q_3_: 4.8–55; range: 1–103), with a median number of publications before residency of 6 (Q_1_-Q_3_: 1.3–39; range: 0–88). The median h-index was 13 (Q_1_-Q_3_: 9–19; range: 2–43). The data for IMGs who completed a research fellowship only vs. those who completed the PhD program is shown in Table 3.

**Table 3:** Data for international medical graduates (IMGs) who conducted research at Barrow Neurological Institute overall and by research fellowship and PhD program participation.

IMG group Metric	Time (months)	Barrow Publications				h-Index^b^
		Total^a^	Before Residency	+1 Year	+2 Year	
All (*n* = 20)						
Mean (SD)	30 (13)	34 (30)	20 (28)	3.1 (3.9)	1.8 (2.1)	14 (8.7)
Median (Q_1_-Q_3_)	24 (24–36)	31 (4.8–55)	6 (1.3–39)	2 (0–6.3)	1 (0–4)	13 (9–19)
Fellowship only (*n* = 17)						
Mean (SD)	26.2 (8.7)	29.8 (28)	17.9 (26.3)	2.5 (3.7)	1.4 (1.8)	13.9 (9.3)
Median (Q_1_-Q_3_)	24 (24–30.5)	30 (3.5–45.5)	4 (0.5–35)	0 (0–3.5)	1 (0–3)	11 (9–17.5)
Fellowship + PhD (*n* = 3)						
Mean (SD)	51.3 (17.2)	56.3 (35.5)	33.3 (40.5)	6.3 (4)	4 (2.7)	17 (4)
Median (Q_1_-Q_3_)	48 (36–70)	58 (20–91)	12 (8–80)	7 (2–10)	5 (1–6)	17 (13–21)

^a^ Total number of publications associated with Barrow after completion of a dedicated study period.

^b^ The Scopus author profile database was used to determine the h-index. Data included publications not associated with Barrow and those published more than 2 years after matching.

## Discussion

### Research demographics of IMGs in neurosurgical residency

Matching into neurosurgery is among the most difficult career choices for USMGs, let alone for IMGs, who face additional scrutiny during the residency matching process. Attempting to set themselves apart, IMGs often invest in years of dedicated research after graduating in their home countries. The median number of months IMGs in our study spent performing dedicated research before entering neurosurgery residency was 30 (Q1-Q3: 18–48). They produced a substantial quantity of published research, with the median number of publications per person being 12 (Q1-Q3: 6–24). The largest proportion of IMGs came from the Middle East (31%); 7% of all respondents were women. Thus, our cohort had demographics similar to the previously described demographics of originating countries and those of the American Board of Neurological Surgery-certified practicing neurosurgeons who are IMGs (6%). Currently, about 19% of US neurosurgical residents are women (17). Many IMGs genuinely enjoy their research activities. Despite the short timeframe to produce a competitive body of work, our results show how valuable IMGs perceive their research experience to be and how much their research experience positively impacted their careers.

### Program director perceptions

This study is the first to investigate neurosurgical residency PDs regarding their views on the value of research as part of the IMG neurosurgery residency application and to compare their expectations with data from successfully admitted IMGs. IMG residency applicants surpassed PD expectations for dedicated research time (12–24 months) and the number of published articles (5). Additionally, we acknowledge that the numbers of peer-reviewed publications do not consider the academic quality, research productivity, or global impact of the published articles. Nor do these data consider the actual contribution of the applicant to the final work (i.e., first author vs. co-author).

Our assessment goes beyond the NRMP Charting Outcomes in “The Match” report (18) that displays the cumulative number of research experiences, abstracts, presentations, and publications included in successfully admitted IMG applications entered in the Electronic Residency Application Service. According to the 2018 NRMP Charting Outcomes in “The Match,” non-US IMGs averaged 3.9 research experiences and 46.6 abstracts, presentations, and publications, whereas USMGs averaged 5.2 research experiences but only 18.3 abstracts, presentations, and publications (19). These data mirror the perceived rise in research productivity of neurosurgery applicants depicted as an “arms race” in the neurosurgery application process (20). Wadhwa et al. commented on a similar trend. They noted the stark difference between the upward trends of the NRMP-reported research numbers and the actual number of peer-reviewed articles published (20). The 5 published articles that are expected of neurosurgery applicants, according to the PDs surveyed, are similar to the previous report of an average of 5.5 publications per neurosurgery postgraduate-year 1 residents in 2018 (20).

These observations suggest that, on average, across all neurosurgery residents, research expectations for future residents are met at the time of application (20). However, other publications that reported data for USMGs and IMGs noted an average of 2 publications by USMG applicants and 5 in the admitted IMG applicant cohort before residency matriculation (11). Given this evidence, it is reasonable to infer that IMGs markedly affect the overall number of publications of medical students who match into a neurosurgery residency program.

To address the qualitative characteristics of an IMG's research productivity instead of absolute numbers, the PD directors were given a 1-question survey at a later time (Table 1). When given the task of ranking research productivity evaluation factors from 1 to 4, most PDs (34/43, 79%) ranked the research institution and its associated personnel as the most important factor. The second most important was the impact of research (i.e., impact factor of journal or h-index). More compelling, all PDs (43/43, 100%) ranked the number of publications as the least important factor. This result brings to light a divergence in the perception of research between IMG and PD that has not been emphasized thus far: the quality of research outweighs the number of publications. This could explain the marked difference in research numbers reported in previous publications, by national databases, and in the present study. Therefore, although an IMG reports a significant amount of research, the data suggests that PDs look beyond the numbers and instead focus on the associated institution and the overall impact and quality of the research work.

### Discrepancy in background and experience between match candidates

An IMG's situation is entirely different from that of a USMG applicant for a neurosurgery residency. Certain IMGs aspire to train in the US for various reasons, including some unrelated to training, such as socioeconomic, political, or quality-of-life motives. They opt to enter research posts and spend years improving their portfolio because it is likely necessary in order to become competitive within the US residency matching system. USMGs complete undergraduate degrees before medical school, prolonging their preclinical and potential research period. In addition, the research opportunities and facilities that US students can access during their undergraduate and postgraduate programs are superior to those of candidates who earn their medical degrees in LMIC countries. According to Sheppard et al. (12), applicants from the top 20 or top 40 US medical schools had higher preresidency publication counts. Applicants with higher preresidency publication counts were also matched at residency programs with highly ranked affiliated hospitals.

In addition to published work, as one of the PDs explained, applicants' research opportunities before the residency match vary greatly, and this variability increases the difficulty of evaluating neurosurgical residency applicants. The importance of an applicant's opportunities is supported by the fact that a significant difference is seen in the number of publications by USMG applicants who graduated from the top 20 medical schools (per *U.S. News & World Report* “Best Research” ranking in 2018), compared to those who did not (9.40 vs. 4.43) (20). In addition, attendance at a top 40 National Institutes of Health–funded medical school was a distinct characteristic associated with successful neurosurgery residency matching (21), with other competitive specialties reporting similar importance of medical school ranking on success in their residency match (22). For example, 40% of US neurosurgery applicants who successfully matched from 2011 to 2018 came from medical schools ranking in the top 40 for research based on the 2018 *U.S. News & World Reports* rankings (12). Furthermore, the opportunities provided by an applicant's medical school are made even more important by a recent change to the United States Medical Licensing Examination (USMLE) Step 1 exam to a pass-fail format. In one study, PDs across all specialties agree that medical school prestige will be considered more important in the evaluation of an application after the change in the USMLE Step 1 scoring format (23). Regarding neurosurgery residency programs, more than half (71%) of the 48 PDs responding to a survey believe that medical school reputation will become more important in resident selection, and 63% of PDs believe it will put IMGs at a disadvantage because of the change (24).

The importance of the research post also translates to the international setting. A higher-ranked medical school seems to similarly impact IMG applicants. In theory, a greater proportion of countries known for their scientific prowess produces more IMGs who are successful in the neurosurgical residency match. The variability of foreign-home institutions, ranging from well-known and research-intensive European centers to those in LMICs with relatively low exposure to scientific work, is represented by applicants (6). In this study, the Middle East accounted for most IMGs in US neurosurgical residency programs (13/42 [31%]). However, about 40% of all Lebanese medical graduates in the past quarter-century have migrated to the United States (25). Arguably this specific example is the exception but again highlights how socioeconomic and environmental aspects, as seen in the Lebanese and Middle East communities, influence international migration. For critical inferences to be made, an analysis of the geographical distribution of all neurosurgical applicants would give a more accurate picture of the country-specific success rates of foreign neurosurgery applicants.

Additionally, while medical school prestige and research opportunities impact a candidate's professional trajectory, elements such as the alignment of foreign medical school curricula to those in the US and collaboration or exchange programs with US institutions, as suggested by Chandra et al. (26), could help explain the observations that specific countries or schools produce more IMGs who successfully enter US neurosurgical residency programs.

### More than a subject of professional competency

The subject of international migration based on professional development is complex and multifaceted. While the US has developed an independent assessment of international professionals, other nations have done the same. Labor ideals, such as free movement and working rights in Europe, have mandated that means be established for assessing international candidates equal to their native counterparts (27). Acceptance of foreign applicants impacts the workforce, resources, and service provision aspects of the neurosurgery profession. Professional immigration has also been the subject of political debate.

The situation for IMGs in the US depends on federal policies, where congressional appropriation for medical training results in appropriate and substantive federal input into the fabrication of residency infrastructure given the government's financial contribution. Graduate medical education programs funded by US tax dollars support the development of the nation's citizens, serve the American public, and USMG and US residency programs. This reality emphasizes the infrastructure's educational, financial, and legislative components to determine the appropriate distribution of resources for training. It provides an infusion of new ideas, rewards for diligent and high-quality professional contributions, professional opportunities, and compatibility with the nation's view of immigration.

The visa requirement to enter and study in the US is a critical component of the neurosurgery residency application process for many IMGs. Because of the length of US neurosurgery training programs, IMGs require a permanent residency permit (“green card”) or other semi-permanent or permanent work authorization. This type of immigration work status can require months to years to acquire, thus impacting the length of an IMG's research period before applying to a neurosurgery residency program.

It is also important to note that professional associations and boards have specific policies regarding training and acceptance. Comparative frameworks include the European and UK systems. Medical graduates of the European Union and other countries with bilateral agreements, such as Switzerland, Norway, and Iceland, are not considered IMGs when applying for residency in these countries. It is particularly important for IMGs to obtain a work permit and accreditation for their medical degrees. This hurdle is comparable to attaining Educational Commission for Foreign Medical Graduates (ECFMG) certification in the US. These accreditations count as the initial basic requirements for practicing medicine in the US.

A “Kenntnisprüfung” in Germany and the Professional and Linguistic Assessments Board (PLAB) in the UK are required to prove sufficient medical knowledge to practice medicine and are only mandatory for foreign graduates (as of January 1, 2021, the PLAB is required of EU citizens) (28). Language requirements, however, are not bound to labor agreements in the European Economic Area due to its multilingual landscape. In Germany, C1 medical language proficiency and B2 German proficiency as per the Common European Framework of Reference for Languages are required (29). This requirement aligns with the Occupational English Test now required by the ECFMG after it canceled the Step 2 Clinical Skills examination for foreign graduates (30).

Furthermore, in the UK, the concept of “experience limits” is a stark difference from the US system of training doctors. In the UK, postgraduate experiences in a medical specialty that exceed certain limits may lead to the status of overqualification for the applicant and ineligibility for residency training. For neurosurgical training, these limitations are as follows: clinical experiences that do not exceed a timeframe of 24 months, with a maximum of 12 months in neurosurgery, neurology, neuroradiology, and neuro-intensive care combined (31). Such a requirement could pose a significant limitation for IMGs if the US had such a directive. This requirement would affect foreign applicants in our cohort, with 5 residents who received some form of foreign training and 5 who completed a neurosurgery residency before entering US residency. In this scenario, IMGs may view the US as an easier or more accessible pathway into postgraduate training programs. Although a maximal preresidency specialty limitation does not apply uniformly to all tracks that may lead to neurosurgical qualifications in the UK (Certificate of Eligibility for Specialist Registration (CESR), CESR-Combined Program), it pertains to the main national training curriculum of an 8-year neurosurgical residency.

The UK exercises annual recruitment for neurosurgical residency similar to the US matching system. As pointed out by Solomou et al. (32), obtaining a neurosurgical specialty training position in the UK was highly competitive in 2018 with 152 applicants for 34 positions and in 2019 with 157 applicants for 24 positions. They noted that proof of early interest in the neurosciences, substantive academic productivity, and undergraduate achievements constituted significant components of a competitive application. Conversely, in Germany, a standardized national process for hiring residents does not exist, and prospective IMGs need to focus on acquiring their work permit and medical accreditation before directly applying to training programs.

The path to residency training for IMGs in the US is well structured. European models appear less well delineated in their residency trajectory. Poorly delineated application processes for achieving professional competency can also hinder international migration and deter potential candidates who favor a more formal approach.

### Benefit of IMG research years beyond their research period

Not only do the research opportunities before the residency application substantially contribute to a successful application, but also factors such as a well-established faculty for mentorship play a role. This point is in line with reports that letters of recommendation (LORs) for the neurosurgical match hold more importance as an admission criterion than the applicant's research, according to PDs (33, 34). Obtaining a recognized, appropriate, or meaningful LOR, which is often as difficult to obtain as for IMGs in their home country, is an additional reason to complete dedicated research time in a US neurosurgical department (35). This time is vital for the advancement of research acumen and ancillary reasons such as producing publications and presentations, attending conferences, networking, building relationships, and time spent as a clinical observer within the research period. IMGs are also evaluated during a research period because the IMG represents an unknown, especially not having been through the standard USMG progression to residency application. These evaluations may include assessments of seriousness, dedication, and persistence—essentially a test of whether the IMG is a good fit for a program. Often, a USMG who does not match may be in a similar situation. Many IMGs enter a US academic environment inexperienced in quality research. Thus, a central question is: “How do IMGs learn research?” Centers should be equipped to offer excellent periods of research where IMGs accomplish research through mentorship and coaching.

Aside from standardized tests for ECFMG certification, it is difficult to determine the quality of international medical curricula and, therefore, the medical education that IMGs receive. In addition, LORs from research mentors abroad are much harder for PDs to evaluate than those from domestic colleagues in the more familiar US neurosurgery community. The time spent on research fellowships in a US neurosurgery department allows an IMG to build rapport with potential future mentors and colleagues and acquire LORs from established US neurosurgeons.

The currently established pathway for IMGs who intend to match into US neurosurgical residency primarily revolves around several dedicated research years. The influences the research years have on the competitiveness of their applications go beyond the quantity of research they produce. Our results suggest that the heavy focus on research might not be warranted or worthwhile in the long term. IMGs often spend onerous time in research that may take on the characteristics of indentured servitude. Thus, the institutional environment must be one of support, mentorship, and positive accomplishment. Information on financial support was not consistent and is variable. Although research project costs are covered, institutions may provide minimal financial living support, requiring the research IMG to establish sufficient personal funds for their stay at the institution. Many institutions now require such individuals to be paid by the institution at levels consistent with National Institutes of Health postdoctoral levels or to establish comparable personal funding, which the US Department of Labor may regulate. IMGs often go into dedicated work positions in laboratories led by a primary investigator working on an established research topic where they are paid from a grant or funded project.

Although most PDs in our survey believed that IMGs produced more work before residency than their USMG counterparts, this impression is not preserved once IMGs enter residency. Although IMG and USMG publication numbers are not available for comparison for the period of neurosurgical residency training, we contrasted the median number of publications by IMGs in residency with those completed before residency (3 articles per year vs. 7 articles per year; Figure 4). This decrease in publications is not surprising because IMG residents are bounded by the same clinical duties and time constraints as USMG residents.

Previous studies found that the academic careers of fellowship-trained vascular, endovascular, and oncological neurosurgeons are primarily associated with the h-index during residency (36, 37). Similarly, Daniels et al. reported the number of publications produced during residency was associated with academic career progression in neurosurgery, whereas the number of publications preresidency was not (38). However, residents who devoted a dedicated research period before their application had better academic career trajectories, but this finding was not differentiated between USMGs and IMGs. However, the input of PDs seems to indicate that the research contributions of IMGs have an impact beyond merely the h-index for their publications.

It is difficult to assess and directly compare USMG neurosurgery applicants who do not spend lengthy dedicated time on research but produce research articles and balance medical school duties with IMGs who work full time on research and do not have clinical responsibilities. In an analysis of burnout in neurosurgical trainees, IMGs score high in resilience (39). A study on general surgery interns found their performance to be of equal quality to USMGs (40). Regarding research, there was no difference in h-indices between IMGs and USMGs during or after residency (7). The analysis of this situation may be more about the assessment of the individual background of the IMG, as more than half of the successfully admitted IMGs had previous neurosurgical training (10/17) in addition to their LORs and US-based research period. IMGs who have experienced previous neurosurgery training may understand the demands that are expected of them and may be committed to seeing their opportunity through to completion. US neurosurgery has had a history of training foreign neurosurgeons who have been successful in academic and private practice environments. Foreign neurosurgeons have also become leaders in American neurosurgery.

The interview process, USMLE scores, and LORs are commonly ranked as more important than research, which supports the sentiments that a well-rounded application of every neurosurgery applicant is of the greatest importance. This finding might encourage future applicants to invest more time in clinical and voluntary work or expand upon their professional connections through neurosurgical meetings and observerships. However, starting at the end of January 2022, the USMLE Step 1 will no longer be graded on a 300-point scale. Instead, it will become graded as pass-fail. A recent study conducted by Huq et al. indicates that most PDs expect the involvement in research and the number of publications to increase among the applicant pool due to the change in the USMLE Step 1 exam format (41). Thus, research performed to gain a neurosurgery residency position may become more consequential.

In addition, barriers are increasing to clinical work for temporary or transient foreign neurosurgeons or medical students in US hospitals due to liability and other legal issues. Clinical or clerkship opportunities are uniquely available to IMGs from Caribbean medical schools due to their location and the fact many are US citizens (i.e., US-IMG) (42, 43). Thus, the research period for IMGs continues to be an opportune means to demonstrate the resourcefulness and accomplishment to support a neurosurgical residency application. With a better understanding of expectations in the most personally controlled component, i.e., a dedicated research period, this study clarifies the cardinal aspect of a successful residency application and how the research period impacts the optimal pathway of IMGs toward neurosurgery residency programs.

### Contributions of Barrow Neurological Institute to IMG development and matching into a US neurosurgery residency program

Another critical question is: “How can the time spent in research as an IMG be made worthwhile?” Several PDs who responded to our survey noted formal neurosurgery department support of IMGs for their research period to be important. One PD cited their American Council for Graduate Medical Education recognition and accreditation for IMG research time. Most PDs in the second survey ranked a candidate's “institution or associated personnel” as the most important factor when considering research productivity. Because their tenure may be years or at least 1 year, involvement of the IMG in a structured, mentored research fellowship or graduate program may be an answer. In 2012, the neurosurgery research laboratory at Barrow (i.e., The Loyal and Edith Davis Neurosurgical Research Laboratory) was a founding member of an integrated interdisciplinary neuroscience graduate PhD program partnership between the hospital institution (Barrow Neurological Institute) and the major local university (Arizona State University), supporting one of the top neuroscience programs in the country. The laboratory supports 2 funded neurosurgery research fellowships per year, with at least 1 one of these positions dedicated to the support of a research fellow in the neuroscience graduate program that lasts from 3 to 5 years, culminating in a PhD in neuroscience. Other research fellows beyond the two positions must self-fund with support verified by hospital human resources administration and be of an amount in line with NIH postdoctoral levels. The laboratory funds all projects, meetings, presentations, and publications costs.

Additionally, neurosurgery research fellows who spend 1 year at Barrow are enrolled in a named research fellowship of the laboratory and institution and receive a formal certificate of research fellowship at the successful completion of their program. They become part of the heritage of the institution, imparting legitimacy to their work and tenure. Thus far, all 3 graduates of the neuroscience PhD program who applied to neurosurgery residency were readily accepted. Furthermore, all 20 IMGs who have worked in the research laboratory since 2000 and applied for residency or a clinical fellowship have been accepted. The duration spent in research by these IMGs is comparable to that reported in the present survey (both 30 months).

The leadership of an in-depth, research-experienced, chair-endowed neurosurgeon engaged full time without clinical duties who directs and coordinates all phases of the laboratory experience, projects, and collaborations, and who skillfully mentors and assesses the research fellows (both IMGs and USMGs) likely plays a major role in applicant acceptance into a neurosurgery residency. A long-established dedicated international outreach toward education in neurosurgery by Barrow's retired and current institutional directors also supports this success.

With a stance similar to Wilder Penfield's viewpoint for training his first research fellows and later residents, [44–46] he and William Cone, and later Arthur Elvidge, allowed trainees and research fellows to develop according to their strengths and interests while providing support and mentorship to help shape their careers. Indeed, attracting research fellows, i.e., IMGs, to become neurosurgery residents is only the beginning. A program director needs to have the skills and resources to inspire these trainees to come into their own. Otherwise, it is a waste of talent and precious career time. Research fellows in the Barrow program are enveloped in a productive environment that focuses on creativity and promotes resourcefulness and innovation.

Interestingly, of our 3 IMGs who were not admitted to a neurosurgery residency program upon the first application, 1 had just arrived in the US a few months earlier and submitted a late, underpowered application. This applicant submitted an excellent application with guidance the next year and was admitted to a prestigious residency. Another elected to continue after 2 years at another institution, at which 2 applications were necessary for residency admittance, without exact details. The third research fellow did not engage in a concentrated or thematic evolution of research but was admitted to a preliminary postgraduate year of surgery and was then admitted to a vacated neurosurgery residency position 2 years later. The common theme of these 3 and the 13 other first-round successfully matched research fellows from our program experience is that all IMG research fellows who were engaged in a structured or thematic line of excellent research, skillfully mentored, and who submitted excellent applications while at Barrow were admitted upon their initial application to a first-rate residency program. Given the success of the IMGs who conducted dedicated research at Barrow, we believe our current model provides a possible solution to the challenges and biases faced by IMGs who desire to train in the US.

### Solutions to possible biases affecting IMG applicants to neurosurgery

IMGs face several institutional biases when applying to a US neurosurgery residency program. These biases result in IMGs being more likely to perform a research fellowship after medical school and matching into an unranked program. In addition, from 2007 to 2019, there has not been a significant increase in the number of IMGs accepted into programs, even though the number of positions has significantly increased and the proportion of IMGs applying has increased.

Although it is beyond the scope of this paper, we believe there are possible solutions to these problems exemplified by our institution. For example, all neurosurgical programs with a dedicated research department or laboratory could foster positions for IMG applicants. This action may even include a graduate program (e.g., PhD program) that extends the IMG's research period and focuses on a specific topic or theme. In our analysis, PDs found this research activity more important than the overall number of publications, which may be on scattered topics or simply one-off clinical papers. Furthermore, these research positions should be more available at highly ranked institutions, which, on average, have more residents per year and resources for performing high-impact research. This outreach may target the bias of not being able to match at a ranked program because from 1968 to 2018, 25% of IMGs who completed a research fellowship stayed at the same institution to complete their residency training (7).

It is somewhat disappointing that only 4 research fellows have been matched into the Barrow residency program, with 2 of them entering vacant positions. Senior staff and faculty believe that the research fellows were not well known by the residents or that the residents do not want to “take a chance” on an IMG in the residency program, perhaps believing they would not fit “the team.” Several research fellows expressed disappointment with not being in the top residency position consideration cohort at Barrow when they had been there for years performing outstanding research or performing a clinical rotation, but where other candidates less familiar and experienced were accepted. These research fellows, however, matched into programs where they stated their research and clinical experience were valued. Fifth-year residents at our institution are critical leaders of future resident selections. Many of our research fellows have won major acclaim for their research work and have already fully trained in neurosurgery at demanding foreign programs, such as Russia's renowned Burdenko National Medical Research Center of Neurosurgery, yet are ranked relatively low. None of the research fellows have had personality issues and have been held in the highest esteem and befriended by department and hospital staff.

Notwithstanding the above, the Barrow fellowship program has achieved success with its structure and reputation. Uniquely, the program has matched 2 foreign fellows in the same year to prestigious residency programs on their initial application, and 2 fellows were matched into the same top residency program over successive years. Although research fellows give presentations, attend rounds, institutional, national, and international conferences, and educational and social events with the residents, solutions to this problem at our institution include further integrating the fellows with residents to apprise them of the full scale of fellows' backgrounds and impending residency applications.

### Limitations

Although our findings portray an interesting component regarding compelling aspects that have evolved in US neurosurgical residency programs, the data presented are limited. These data concern IMGs in US neurosurgery training programs only. The response rate to surveys was about 50% for IMGs and 28% for PDs; thus, it is reasonable to argue that the opinions obtained do not accurately represent the complete resident demographic and PD opinions and policies. Our data do not derive from social media or publicly available databases, which may yield large numbers but few personal details. However, the data are sufficient. Our sample size is sufficient considering the actual size of the denominator (78 or 79). First, a search before and after residency graduation in June 2020, revealed 8 residency classes and 79 IMGs. Second, after collecting archived data from the NRMP from 2013 to 2020 (8 years), we found that 95 IMGs had been accepted into US neurosurgical residency programs, of which 78 were non-US IMGs. Our study only surveyed non-US IMGs. Lastly, the 79 IMGs (from before and after resident graduation) and the 78 non-US IMGs (from the NRMP archive data) do not accurately represent the number of non-US IMGs who conducted dedicated research as fellows before applying to US neurosurgery residency programs. This number cannot be accurately determined but is less than 78 or 79. Therefore, our already high response rate (53%) for non-US IMGs who successfully matched into US neurosurgery residency programs most likely represents an even more significant percentage of non-US IMGs who conducted research through a fellowship and were accepted into a US residency program, perhaps as high as 80%. In addition, no previous study has included information from neurosurgery residency PDs.

Respondents were cautious with some of their responses to questions (i.e., country of origin and number of publications during residency). As such, the data might not be representative of the cohort due to a lack of responses from some residents. The present paper only describes the results regarding IMG research productivity, and it does not address all the questions given in the questionnaires. Other survey results will comprise additional studies. We used as much accessible NRMP and published literature as possible for comparison. In cases where similarities were identified, the findings suggest the continuity and the legitimacy of the representative trends.

Furthermore, some important questions cannot be addressed by the present study but merit answers. Is the neurosurgical training system in the US the best in the world? If so, does it allow weaker candidates to become excellent neurosurgeons? Is the international community lacking excellent potential neurosurgeons? Regarding the PD role, do PDs require qualities (i.e., aptitude, passion, predisposition, teamwork, or ambition) that cannot be evaluated through an IMG's research productivity? Is a research fellowship necessary for an IMG, or could an IMG and USMG apply under the same conditions? Is neurosurgical attitude associated with the prestige of the home institution of a candidate? Lastly, how can the system be improved to offer all IMGs a similar condition when applying? All these questions are critical and, unfortunately, outside the scope of the present study due to its strict focus on the IMG research period from the perspective of both IMGs and PDs. We acknowledge that a focused editorial on such a topic would be justified.

## Conclusions

The IMGs surveyed reported significantly longer periods invested in dedicated research and more published articles before their US neurosurgery residency match than the expected numbers reported by the PDs surveyed. PDs perceive IMGs to be more productive in their research than USMGs until they enter residency. At that time, many PDs stop seeing a difference in research productivity between IMGs and USMGs. This impression is in accordance with our finding that there is an understood decrease in published work by IMGs upon entering residency. Many IMGs complete a dedicated research period in a US institution before their residency application, unlike USMGs. This dedicated period allowed them to surpass the research productivity expectation of PDs and enhance their neurosurgery residency application. This study highlights that the research requirement is more than satisfactorily achieved by IMGs, and to improve their competitiveness, IMGs may be better served by completing more clinical placements. However, with limitations in clinical positions and neurosurgery's tradition of research involvement, neurosurgery residency training in the next decade will be defined as much by advances in technology as by the opportunities afforded in neurosurgery training and the labor shifts in the overall profession. As neurosurgical education and technology advance worldwide with growing interconnections between neurosurgeons of different countries, potential changes in the requirements and policies of training programs may open training positions for successfully and comparatively educated IMGs in various countries.

In 1928 when he arrived at McGill University's Royal Victoria Hospital under the aegis of Edward Archibald, Wilder Penfield pioneered what would be a remarkable achievement 6 years later with the opening of the Montreal Neurological Institute. He sought nothing in the way of nationalism, only a pursuit of excellence—his first research fellows arriving in 1929 were from San Francisco and London, with one woman—the future famed neuropathologist Dorothy Russell—and his residents were as well international, contributing brilliantly in the next decades to scientific neurosurgery (44, 45). In fact, up until the mid-1990s, McGill had trained more department chairmen of US neurosurgery programs than any other single institution (46). McGill trained the first African American neurosurgeons during a period of intense racial segregation in the US, “enabling subsequent African Americans to enter and enhance the field of neurosurgery.” Up until 1997, a unique, close relationship existed between American and Canadian neurosurgery since famous institutions of the two countries were among the founding centers of neurosurgery in North America, with activities, training, faculty, and programs constantly shared. Unfortunately, unresolved training, practice, and economic issues since have designated Canadians as ordinary IMGs to the US neurosurgery system. For many years, Americans went abroad for research at various times related to their residency period, although most returned to the US for clinical training. Although the notion of protecting national interests is crucial and socioeconomic attractions are a powerful attractant to the US medical practice environment, might we take an altruistic lesson from Penfield, that excellence and the deserving, no matter what human form, are just as critical to the progress and improvement of neurosurgery and its training milieu. A delicate balance is also necessary between the workforce and national interests. We hope that our findings will benefit future applicants and PDs alike and encourage further investigation of IMG applicants to neurosurgery training programs.

## Data Availability

The raw data supporting the conclusions of this article will be made available by the authors, without undue reservation.
